# The Tibetan Uterotonic Zhi Byed 11: Mechanisms of Action, Efficacy, and Historical Use for Postpartum Hemorrhage

**DOI:** 10.1155/2012/794164

**Published:** 2011-07-24

**Authors:** Rebecca Lynn Coelius, Amy Stenson, Jessica L. Morris, Mingji Cuomu, Carrie Tudor, Suellen Miller

**Affiliations:** ^1^School of Medicine, University of California, San Francisco, 50 Beale Street, Suite 1200, San Francisco, CA 94105, USA; ^2^Department of Obstetrics and Gynecology, David Geffen School of Medicine, Center for the Health Sciences, University of California, Los Angeles, 10,833 Le Conte Avenue, Los Angeles, CA 90095, USA; ^3^Safe Motherhood Program, University of California, San Francisco, 50 Beale Street, Suite 1200, San Francisco, CA 94105, USA; ^4^The Institute for Social and Cultural Anthropology, University of Oxford, 386 London Road, Headington, Oxford OX3 8DW, UK; ^5^Tibetan Medical College, Lhasa, Tibet 850000, China; ^6^School of Nursing, The Johns Hopkins University, 525 N. Wolfe Street, Baltimore, MD 21205, USA; ^7^Department of Obstetrics, Gynecology, and Reproductive Sciences, University of California, San Francisco, 50 Beale Street, Suite 1200, San Francisco, CA 94105, USA; ^8^Safe Motherhood Programs, Bixby Center for Global Reproductive Health, School of Medicine, University of California, San Francisco, 50 Beale Street, Suite 1200, San Francisco, CA 94105, USA; ^9^Maternal Child Health Program, School of Public Health, University of California, Berkeley, Berkeley, CA 94720, USA

## Abstract

*Objective*. To explore evidence for the traditional Tibetan medicine, Zhi Byed 11 (ZB11), for use as a uterotonic. *Methods*. The eleven ingredients in ZB11 were chemically analyzed by mass spectroscopy. A review was conducted of Western allopathic literature for scientific studies on ZB11's individual components. Literature from Tibetan and other traditional paradigms were reviewed. *Results*. Potential mechanisms of action for ZB11 as a uterotonic include laxative effects, a dose-dependant increase in smooth muscle tissue peristalsis that may also affect the uterus smooth muscle, and chemical components that are prostaglandin precursors and/or increase prostaglandin synthesis. A recent RCT demonstrated comparable efficacy to misoprostol in reducing severe postpartum hemorrhage (PPH) (>1000 mL) and greater effect than placebo. Historical and anecdotal evidence for ZB11 and its ingredients for childbirth provide further support. *Discussion*. ZB11 and its ingredients are candidates for potentially effective uterotonics, especially in low-resource settings. Further research is warranted to understand the mechanisms of action and synergy between ingredients.

## 1. Introduction

### 1.1. Uterotonics for Prevention of Postpartum Hemorrhage (PPH)

PPH is a leading cause of maternal morbidity and mortality worldwide. It is estimated that of the approximately 350,000 women who die annually from complications of pregnancy and childbirth, more than 25% die of obstetric hemorrhage [1]. This burden is unequally held by developing countries, where it occurs at a rate 100 times higher than in the developed world [2]. One key factor is that many women deliver at home without skilled delivery attendance, where complications often go unrecognized and untreated. By the time a problem is identified and the woman is transported to an appropriate facility, it may be too late. Women can deteriorate so rapidly that even if they arrive at an appropriate medical facility alive, they may already be in irreversible shock and/or have developed disseminated intravascular coagulopathy (DIC) [3]. 

The third stage of labor is the time period between the birth of the infant and delivery of the placenta and membranes. Failure or delay of the uterus to appropriately contract after delivery can lead to rapid and massive hemorrhage. Shortening the third stage of labor and ensuring that the uterus is well contracted during this time has the potential to decrease blood loss and the incidence of hemorrhage. The World Health Organization (WHO), the International Federation of Gynecology and Obstetrics (FIGO), and the International Confederation of Midwives (ICM) advocate the use of a uterotonic to decrease postpartum bleeding by up to 50%–70% [4]. 

 A uterotonic is a substance that increases the tone (causes contraction) of the uterus, an organ composed of smooth muscle tissue. In both allopathic and traditional and herbal medicine, substances that are called uterotonics often have laxative, purgative, diarrheagenic, cathartic, abortifacient, and emmenagoguic effects. Some uterotonics are biochemically synthesized hormones, such as oxytocin, that act on distant hormone receptors or upstream from other hormones in the body to induce uterine contractions. Others may be synthetic prostaglandins or prostaglandin precursors. Prostaglandins are lipid compounds derived enzymatically from fatty acids and serve as locally acting messenger molecules performing important functions in the body such as regulating the contraction and relaxation of smooth muscle [5]. 

Since 2007, the WHO PPH Prevention Guidelines have stated that the uterotonic of choice for prophylaxis of PPH is 10 IU of oxytocin delivered intramuscularly [6]. Oxytocin (pitocin and syntocinon) is a hormone produced in the hypothalamus that plays a critical role in labor and delivery by stimulating uterine contraction, and in lactation by causing milk letdown. However, there are barriers to its use in low-resource settings. To maintain the highest potency, oxytocin requires refrigeration. It is only effective if given parenterally [4], thus safe administration of oxytocin requires staff trained in intravenous or intramuscular administration techniques, sterile needles, and safe disposal for injection equipment. These are frequently unavailable or too costly during births in low-resource settings. The uterotonic ergometrine has similar efficacy to oxytocin but has more side effects, which makes it the preferred option only when oxytocin is not available [6]. Like oxytocin, its utility in low-resource settings is lessened by special storage requirements and parenteral administration [7]. 

The uterotonic misoprostol has been recommended as an alternative to oxytocin and ergometrine for the prevention of PPH in low-resource settings, primarily due to its greater ease of administration and storage [8]. Misoprostol (Cytotec) is a synthetic prostaglandin E1 analogue that has been shown to significantly decrease PPH when compared to placebo [9, 10]. Unlike oxytocin, misoprostol can be taken orally and is also relatively stable; it can be stored at room temperature in a closed container safe from humidity with a shelf life of several years [11]. 

Misoprostol has drawbacks, most notably side effects including pyrexia, nausea, shivering, and diarrhea. Studies of 600 mcg of misoprostol have shown pyrexia and shivering to be most common, with 4%–38% reporting pyrexia and 32%–57% reporting shivering [7, 9, 10]. These findings are dose dependant; malignant hyperpyrexia has been reported at 800 mcg orally [10, 12], with potentially dangerous outcomes such as uterine rupture, abruption, fetal demise, and maternal death if used inappropriately during the active phase of labor at doses above 50 mcg [13, 14]. Another barrier is that misoprostol is not approved for obstetric or gynecologic indications in many countries [15], and thus may not be widely available. Where use is restricted because of concerns for its use as an abortifacient, it may only be found on the black market, causing concern for its purity and safety. Due to misoprostol's side effects and inconsistent outcomes across studies [16], the search for an alternative oral uterotonic for low-resource settings continues. 

The ideal uterotonic for prevention of PPH at the home or community level is one that is simultaneously efficacious, affordable, widely available, does not require electricity or technology for effective storage, has a low side effect profile and high safety level, and is compatible with local beliefs and traditional practices. There may be alternatives to current allopathic medicines within traditional medical systems that would fit these ideals for the prevention of PPH. 

Since at least 1500 BC traditional medical practices have taken advantage of the uterotonic properties of local herbs to promote uterine contractions and/or control PPH. These systems include but are not limited to US-based midwifery, African traditional, Ayruveda, Chinese traditional, and Tibetan medicine. The history of ergometrine is an example of how a naturally occurring substance was discovered to be a uterotonic and became widely used in Western allopathic medicine. Ergometrine is derived from the naturally occurring substance ergot, the alkaloid-containing product of the fungus, *Claviceps purpurea*, which grows on grain. References to ergot date as far back as 600 BC, and its effects on pregnancy were identified in the 16th century by midwives who noted an increased rate of miscarriage during epidemics of “ergotism”, a disease caused by eating contaminated rye bread [17]. In 1935, the active substance was identified and named ergometrine, effectively initiating the modern era of allopathic uterotonics [18, 19]. 

In the USA, Chumash Native American communities in California have used *Trichostema lanatum* and *lanceolatum* leaves in a decoction to pass the afterbirth (placenta) [20]. Midwives frequently use herbal preparations that are purported to increase uterine tone (red raspberry leaf), act as an emmenagogue to provoke menstruation (penny royal), or induce labor contractions (blue cohosh, black cohosh, and castor oil) [21]. Among South African native populations, the Xhosa, Zulu, and Sotho tribes use over 57 traditional herbal medicines during pregnancy and childbirth [22], which have exhibited direct smooth muscle activity on the uterus and/or ileum in vitro [23]. In Ayurvedic medicine, the gentle anthraquinone-containing laxative Aragvadha, *Cassia fistula-Fructus, *is used in pregnancy to stop bleeding and cause a general “downward” movement. Rhubarb root is administered internally to treat many forms of hemorrhage [24]. In traditional Chinese medicine, (TCM), Motherwort, or *Leonurus heterophyllus sweet* is considered the key ingredient for PPH prescriptions and has been developed into an injection for clinical use (personal communication with Taxiang Wu, Chinese Cochrane Centre, December 10, 2009). 

In the Tibetan Autonomous Region (TAR) of China, Zhi Byed 11 (ZB11) has been the traditional treatment of choice for PPH for over 700 years and continues to be widely used by Tibetan traditional healers and providers. It is also prescribed to aid and quicken delivery, increase uterine contractions, deliver the placenta, and to manage other complications of pregnancy such as hypertension and infection. Currently, much cheaper than conventional uterotonics at USD 0.04 per dose, ZB11 is also widely available in the TAR and has a high degree of acceptability. Taken orally and not requiring any special storage, ZB11 is practical for home and community births. ZB11 seems to hold none of the barriers allopathic uterotonics such as oxytocin, ergometrine, and misoprostol do for use in the TAR, and continues to be used by Tibetan traditional healers [25].

Phuntsog Wangmo, a traditional Tibetan healer, and the cultural director and an instructor at the Shang Shung Institute of America, International Institute for Tibetan Studies, Conway, Mass, USA, shares her personal experience using ZB11 in contemporary rural Tibet:

“Zhi Byed 11, we use this commonly, this is a very good medicine, it helps to clean the uterus, for example, it helps if something remains in the uterus to make sure that it is expelled. When I am working with someone in labor, I do several things. I will try to make sure that the baby's head is down…. We give some nutrition (like warm broth) if the person is strong enough. If contractions are getting weak, or the patient does not dilate, we give ZB11. We give one pill orally. If nothing happens after 1.5 hours, we can give another one. We are allowed to give 2-3 pills. Once the baby has come out, we give warm oil treatment. We keep the mother warm and give her nutritional food, warm broth or soup. At the same time, we get the placenta, put oil on the abdomen, and do gentle massage, downward traction on placenta. If the placenta does not come out or if some pieces remain inside, we would give another ZB11. We watch the bleeding, if it continues, we check the color of the blood. If dark, we are not so worried, if it is fresh, then we are worried. I worked in a remote area, so I have to stay with them at their house. If needed, I stay overnight. If I have to leave, then I give a good dosage of Gur gum 8 and make sure no bleeding. I leave them with 3–5 pills of ZB11, and have them take 1 in the morning for three days. The reason for this is to clean the uterus and keep it healthy. I think it works as a team between all the ingredients. The contractions become stronger, more painful when it is given. I use it in almost everyone, but the amount varies, between 1 pill or many pills. I was always able to get that medicine, the ingredients are not that difficult to find. It is not expensive and there are many good sources.”

**Figure 1:**
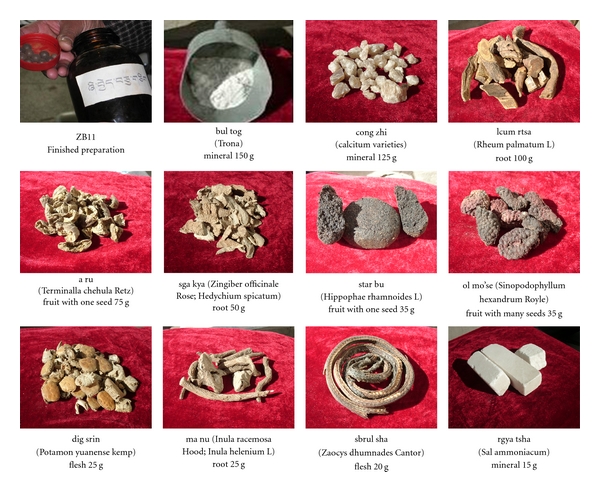
Zhi Byed 11 ingredients by name, component, and weight.

Traditional Tibetan medical literature states that ZB11 owes both its efficacy for preventing and treating PPH, and low rate of side effects, to a careful balance of eleven synergistic natural ingredients (see Figure 1). Recently, ZB11 became the first traditional uterotonic to be rigorously tested against an allopathic uterotonic for PPH. In a double-blind, randomized, controlled trial (RCT) conducted by Miller et al. [25] in three maternity hospitals in Lhasa, TAR, delivering women (*n* = 967) were randomly assigned to receive either ZB11 or misoprostol [25].

As herbal products can differ depending what part of the ingredient is used, the ratios, and the quality of the ingredients, the Miller et al. [25] study used a single large batch of ZB11 made at a local factory, the the Mentzikhang Traditional Tibetan Medicine Factory, in Lhasa, Tibet in May 2004. Employing only one factory and using one single batch for the entirety of the study minimized this risk. Samples of the study's ZB11 product were retested for potency every six months throughout the trial from August 2005 to March 2007 to confirm there was no degradation. 

The study's primary combined outcome, which included incidence of PPH (blood loss ≥ 500 mL) administration of open label uterotonics, or maternal death, was found to be lower among the misoprostol group (16.1% versus 21.8% for ZB11; *P* = 0.02). The proportion of women who experienced blood loss ≥ 500 mL was also lower with misoprostol (12% versus 17% ZB11). However, there were no significant differences in the more clinically important indicators of PPH: blood loss ≥ 1000 mL, (2.1%, misoprostol versus 3.1%, ZB11, *P* = 0.29), mean measured blood loss (304 mL for misoprostol versus 332 mL for ZB11, *P* = 0.15) or median measured blood loss (250 mL versus 265 mL for ZB11, *P* = 0.086). The length of the third stage of labor was also similar (7.2 min for misoprostol versus 6.9 min for ZB11). Use of additional uterotonics occurred in 16% of those given ZB11 and 13% of those given misoprostol. Side effects were more common in the misoprostol group, with fever significantly more common in the misoprostol group (2.7% versus 0.8%, *P* = 0.03). There were no maternal deaths in either arm of the study [25].

ZB11's comparable results for several outcomes, long history of use and acceptability in the TAR, strong anecdotal support from Tibetan healers, low side effect profile, and the pressing need to find a uterotonic appropriate for use in low-resource settings prompted us to take a more detailed examination of ZB11. We were specifically interested in ZB11's individual ingredients and any known synergistic effects between ingredients for possible mechanisms of action as a uterotonic, their toxicology and safety profile in clinical use, and historical and current uses, especially for purposes related to childbirth.

## 2. Methods

In this paper, we explore the potential mechanisms of action for ZB11 as a uterotonic, either as a combined preparation or as a subset of its individual ingredients. The methodology of this paper makes use of chemical analysis, evidence-based review of both Western allopathic literature and peer reviewed journals on traditional and herbal medicine, and a review of Tibetan and other traditional and herbal medicine paradigms.

The chemical makeup of the ZB11 used in the Miller et al. [25] study was examined by mass spectroscopy at the University of Utah Center for Human Toxicology [27]. These findings were then cross-checked against those reported for each ingredient in *Mosby's Handbook of Herbs and Natural Supplements* [28].

A literature search was conducted for each of ZB11's eleven ingredients. Databases used included PubMed, the Cochrane Database of Systematic Reviews, and the CENTRAL Controlled Trials Database. No parameters were set for year, as many herbal medicines have a long history and historical accounts may be considered valuable. The terms entered were both the Latin name and the common name of the individual ingredients found in ZB11. Articles in English or those translated into English were examined. We reviewed articles and selected those with relevance to potential mechanisms of action on smooth muscle (as the uterus is made up of smooth muscle), pregnancy, bleeding, or uterotonic function. All animal and/or human studies that used the pure ingredients were included. Information about side effects and toxicity was also collected from this search. The historical usage of ZB11 as a complex preparation of eleven ingredients and of each individual ingredient was explored by a review of texts on Tibetan traditional medicine, traditional Chinese medicine, and herbal medicine. 

## 3. Results

### 3.1. Chemical Analysis

The ZB11 used in the Miller et al. [25] study was examined by mass spectrometry by Andrenyhak [27], University of Utah Center for Human Toxicology. This paper was created to test the ZB11 product to be used in the Miller et al. [25] study. The primary compounds identified included long chain fatty acids, long chain fatty alcohols, and long chain methyl esters, such as Eudesma (5,11 (13)-diene-8,12-olide), Physcion, and Chrysophanol (9,10-Anthracenedione, 1,8-dihydroxy-3-methyl) (see Table 1) [27].

### 3.2. Review of Western Allopathic Literature

Both RCTs and animal models were found for four of the ZB11 ingredients which suggested possible mechanisms of action for *lcum rtsa, star bu, *and *sga skya *as uterotonics (see Table 2). These include the laxative effects of anthranoids, smooth muscle contractions of emodins (dose dependent), the peristaltic effect of sennosides on the gastrointestinal system, and the prokinetic and drug potentiating activity of *sga skya* (due to its inhibition of first pass hepatic degradation). While no studies looked specifically at the uterus for mechanism of action, the effects on smooth muscle and the GI system is relevant, since the uterus is also made up of smooth muscle. Thus, often these mechanisms affect multiple organs with smooth muscle and similar receptors, as evidenced by some of the side effects experienced by the GI system when allopathic uterotonics are used. Sga skya's potentiating mechanism would make any ingredient processed by the liver more potent and/or act longer though we were not able to find studies documenting whether the liver is responsible for the metabolism and excretion of other ZB11 ingredients. Conversely, *ma nu* was found to have smooth muscle relaxant activity on the ileum and trachea; there was no study documenting its effect on uterine smooth muscle.

Typical use of ZB11 is a short course (1–3 days) at relatively low doses (1–3 pills.) In the Miller et al. [25] study, all side effects, including diarrhea, shivering, and fever, were more common in the misoprostol group than the ZB11 group, with fever being significantly higher (2.7% misoprostol versus 0.8% ZB11, *P* = 0.03). While this is a statistically significant result, clinically, it may not be considered relevant. Shivering was the most common side effect, occurring in 12% of participants given ZB11 and 16% of participants given misoprostol. There were no life threatening adverse events reported for ZB11 in this trial [25].

Other studies also describe side effects associated with some ZB11 ingredients (see Table 3). It is important to note that outside the Miller et al. [25] study, these reported side effects were not accompanied by specific dosage levels or schedules. The most serious side effects were reported only anecdotally with unquantified long-term use or high doses and were not reported in any controlled trials or animal models. [25, 33].

### 3.3. Review of Traditional and Herbal Medicine Literature

There was strong historical and anecdotal evidence for the use of ZB11 ingredients, not only in Tibetan medicine (see Table 4) but also in other traditional medicines (see Table 5), as diuretics, uterine, and other smooth muscle stimulants, laxatives, and for side-effect management such as preventing and treating nausea and vomiting in a range of disorders. 

## 4. Discussion

Our analysis supports ZB11's safety and effectiveness as a uterotonic with the potential to decrease the risk of PPH, particularly in low-resource settings, where current allopathic uterotonics face significant barriers to use. ZB11 has several qualities that make it an attractive uterotonic for prevention and/or treatment of PPH at the home or community level in Tibet. With over 700 years of history, it is widely culturally accepted by birthing women, Tibetan healers, and family members in Tibet. At the same time, it is highly affordable (USD 0.04 per dose) widely available, does not require electricity or technology for effective storage, and requires minimal training for administration. Its safety profile is similar to misoprostol, the current standard of care. In places where access to a steady supply of “Western” medication may be limited, this offers an important, less costly alternative.

Several possible mechanisms of action were identified for ZB11 as an uterotonic. These include a dose-dependant increase in peristalsis of smooth muscle, which is the uterus tissue type. Fatty acid esters, such as the linoleic acid, methyl ester, and oleic acid methyl ester found in the mass spectrometry solution of ZB11, are prostaglandin precursors and are involved in prostaglandin synthesis [45, 46]. Eudesma, or *Inula helenium*, contains alantolactone, which is known to have cathartic, or purgative properties (emmenagoguic, secretagogue, secretolytic) [47]. 9,10-Anthracenedione, 1,8-dihydroxy-3-methyl (also known as chrysophanol) and rhein are both anthroquinones found in Chinese rhubarb species, such as *Rheum palmatum*. Researchers at the Beijing Medical University's College of Pharmacology have conducted studies on the chemical composition and bioactivities of dozens of *Rheum* species and attribute many with purgative properties. Rhein and chrysophanol are also found in the senna species, known as a laxative [25, 33].

In the Miller et al. [25] study, the sample size was calculated using the assumption that the effect of ZB11 would not be the same as placebo but would be at least a 10% decrease from hemorrhage occurrence when no treatment was given. This assumption was validated in the study, where there was a 15% decrease in PPH rates from baseline (before the initiation of any treatment.) This 15% decrease further supports the uterotonic effect of ZB11. Notably, in the same study, there was no significant difference between misoprostol and ZB11 in blood loss ≥1000 mL. This is striking given that blood loss ≥1000 mL is considered one of the most clinically meaningful indices of PPH, correlating with the 95th percentile for blood loss and greater associated morbidity and mortality [48]. An uncomplicated vaginal delivery often results in blood loss greater than 500 mL. A change in systolic blood pressure is not seen until 1000 mL is lost, and a blood loss of greater than 2000 mL is necessary before systolic blood pressure reaches levels as low as 50–70 mm Hg [49]. More research is warranted to determine the true effect of ZB11 in preventing and/or treating PPH. Given that a placebo-controlled trial is unlikely due to ethical challenges, the best option may be a double-blinded noninferiority trial of ZB11 versus misoprostol, with the main outcome evaluating the effect on severe hemorrhage (blood loss > 1000 mL). We also recommend that any future RCT follow the CONSORT statement for herbal medicine RCTs [50].

Traditional Tibetan medicine is crafted for each patient depending on many specific environmental and individual factors. Scientifically, this is difficult to evaluate using a “Western” approach. Similar to dosing trials in the pharmaceutical industry, different dosages and/or combinations of ZB11 ingredients may alter the efficacy and/or side effects of the medication. Evaluating different combinations of all 11 ingredients would require a large amount of resources and time and still may not result in a “one size fits all” pill. Therefore, capturing the true effect of ZB11 via standardized scientific “Western” analysis may always be difficult. It may be useful to conduct more qualitative work surrounding the traditional proportions of ingredients that make up ZB11 and test a wide range of samples to identify the most common mixture though a challenge is that traditionally a mixture is made specific to each patient. It may also be beneficial to compare different formulations of ZB11 looking for a balance of efficacy and safety before beginning a trial.

The reported side effects in the Miller et al. [25] study of diarrhea, shivering, and fever were lower for ZB11 than with oral misoprostol though the clinical significance of the difference is unclear. If a medication has a high side-effect profile, medical personnel may be reluctant to administer the medication; women themselves may refuse to receive it in subsequent deliveries or may tell friends and family to avoid the medication and/or the facility where it is administered. Several of the ingredients in ZB11 are known in Tibetan Medicine to prevent the most common side effects that typically result from administration of prostaglandins, and were seen at lower rates with ZB11 than with misoprostol in the Miller et al. [25] trial. For example, *cong zhi* is used to prevent and/or treat gastrointestinal side effects; *ma nu* prevents fever, *sdig srin *prevents cramping, and *a ru* is generally believed to increase body energy and prevent a host of side-effects [33, 34, 37]. It may be that the lower side effect profile of ZB11 is due to these balancing effects although this has not yet been critically analyzed. 

Historical and anecdotal evidence for ZB11 and its ingredients' clinical utility provides further support for ZB11's potential efficacy. What is especially interesting to note is that some of ZB11's ingredients are used for similar purposes across very different healing traditions. In African herbal medicine, *bul tog* (*calcium bicarbonate)* is a well-known laxative [40], while in Western Uganda, *Sga skya,* (*Zingiber officinale rose) *is used to induce contractions [22]. *Ol mo'se (Himalayan mayapple) *is used in traditional Chinese medicine as a strong uterine stijmulant [26]. Rhubarb derivatives, such as *lcum rtsa*, also appear in systems as diverse as traditional Chinese and Aryuvedic medicine to treat hemorrhage [21, 42]. The use of similar ingredients to augment labor and cause uterine contractions in diverse traditional medical practices lends support to the probability of an uterotonic effect. While one explanation may be that these healing traditions stem from similar ancestry and heritage, the persistence of these compounds still suggests that they have been found in practice to be effective by healers over many generations. Evaluation of these compounds and others within traditional medicine has the potential to identify culturally relevant, effective regimens that may prevent morbidity and mortality from obstetric hemorrhage. 

A limitation of our study was that we only looked at three databases, none of which were Chinese (such as Chinese National Knowledge Infrastructure and Wan Fang) due to language barriers). We were also only able to review papers that were written in or translated to English. Thus, studies may be missing from our analysis. Moving forward, we recommend that the database sources be broadened, and that additional Tibetan healers be consulted for their personal opinions on and experience with ZB11.

## 5. Conclusions

Progress in Western medicine has often been the result of accidental discovery, creating synthetic versions of naturally occurring plant and animal substances, or adaptations of a traditional medical system's preparations. Examples include the uterotonic ergometrine's derivation from the fungus *Claviceps purpurea*, and the use of the ancient Chinese Traditional medication, Qinghao (*Artemisia* annua) for the modern treatment of malaria. The findings of this ZB11 review suggest potential for both allopathic and continued traditional use of ZB11 as an uterotonic, with further research warranted to understand the underlying mechanisms of action and synergy between ingredients.

##  Conflict of Interests

None of the authors has any conflict of interests to disclose.

## Figures and Tables

**Table 1:** Zhi Byed 11 Tibetan, Latin, and common names, chemical components.

Tibetan name	Latin name	Common name	Chemical component [25, 26]
a ru	Terminalia chehula retz	Chebulic myrobalan	Ellagic acid, gallic acid, lufeoic acid, and chebulinic acid

bul tog	Trona	sodium, bicarbonate	NaHCO_3 _

cong zhi	Calcitum	calcium	Calcium

lcum rtsa	Rheum Palmatum L	Chinese, Turkish, or East Indian rhubarb	Anthraquinones: rhein, chrysophanol, aloe emodintannins gallo, galloy-1glucose stilbene, phenolic, and polyketide synthase sennosides

ma nu	Inula Racemosa Hood, Inula Helenium	elecampane	Volatile oil: alantolactone lactone: Alantol polysaccharides: lnulin

ol mo'se	Sinopodophyllum hexandrum (Royle)	Himalayan or Chinese mayapple	Podophyllotoxin Picropodophyllin: Apha and beta phyllin

rgya tsha	Sal ammoniacum	salt of sulphur/tar	Na_2_S

sbrul sha	Zaocys dhumnades Cantor	black snake meat	Not found

sdig srin	Potamon ynnanense kemp	freshwater crab shell	Not found

sga skya	Zingiber offficinale Rose, Hedychium Spicatum Ham	ginger, ginger Lily	Pungent gingerol, zingerone, Shogaol volatile oil, bisabolene, zingiberene, zingiberol, and Sesquiterpene

star bu	Hippophae rhamnoides L	sea buckhorn	Anthranoid, emodin, anthraquinone glycosides, frangulin A, B, glucofrangulin A, B

**Table 2:** Potential mechanisms of action of Zhi Byed 11.

Tibetan name^a^	Mechanism of action
lcum rtsa	Anthranoids: laxative due to direct colon irritation, purgative potentially due to abnormal expression of aquaporins [27].
	Emodins: dose-dependant smooth muscle contraction [28] due to increased concentration of calcium in smooth muscle and the activation of calcium chloride channels via enhancement of membrane Gi/Go protein signal transducer pathway [29]. CaCl_2_ can reverse effects. Heightens effects of Acetylcholine [28].
	Sennosides: animal studies demonstrate intestinal peristalsis due to depolarization of cell membranes, quickened burst slow wave potential, and increased frequency of spike potentials [30].

ma nu	Highly potent tracheal and ileal smooth muscle relaxant in animal studies [30].

sga skya	Decreases nausea and vomiting [31] and prokinetic activity in digestive system of animal models due to effects on muscarinic M3 receptors of stomach fundus [32].

star bu	Anthranoids: Laxative due to direct colon irritation [19].

^a^Studies on *sbrul sha*, *ol mo'se*, *rgya tsha*, *a ru*, *bul tog*, *cong zhi*, and *sdig srin* are not found.

**Table 3:** Zhi Byed 11 ingredient side effects and toxicities.

Tibetan name^a^	Common side effects and toxicities [32, 33]	Side effects and toxicities in large doses/prolonged use^b^ [33]
cong zhi	Constipation, anorexia, nausea, vomiting, flatulence, diarrhea, and rebound hyperacidity	None found
lcum rtsa	Cramp like discomforts	None found
ma nu	Irritate mucus membranes, sensitizing, and can cause allergic contact dermatitis by forming haptens	Vomiting, diarrhea, cramps, and symptoms of paralysis
ol mo'se	Nausea, vomiting, diarrhea, and abdominal pain	Seizures, stupor, coma, hepatotoxicity, leukopenia, thrombocytopenia, anemia, and apnea
sga skya	Nausea, vomiting, anorexia, and hypersensitivity reactions	Arrhythmias
star bu	Nausea, vomiting, diarrhea, anorexia, abdominal cramps, hepatotoxicity, dehydration, and nervousness/tremors	None found

^a^ *sbrul sha*, *rgya tsha*, *a ru*, *bul tog*, and *sdig srin* toxicity data are not found ^b^ *exact dosage not stated, but reported as “large” or dosing “prolonged”.*

**Table 4:** Uses of Zhi Byed 11 Individual Ingredients in Traditional Tibetan Medicine.

a ru	Laxative [34], improves and gives positive body energy, helpful for most diseases.
bul tog	Laxative, synergy with lcum rtsa; downward expelling [34]
cong zhi	Prevents vomiting, treats constipation [35] balances bul tog by absorbing carbonic acids [34]; treats diarrhea caused by either virus or indigestion; supports “bon” nutrition; helpful for treating fever from the endocrine system and for treating ulcers [36]
lcum rtsa	Treats irregular menstruation [33]
ma nu	Induces sweat and treats fever [36]; downward expelling functions [33]
ol mo'se	Thins coagulated blood, promotes normal menses; promotes uterine contractions, delivers baby, expels placenta, some consider the most important ingredient in *ZB11 *[33]. Also used to cleanse other uterine disorders [36]
rgya tsha	Promotes uterine contractions, dilates cervix [33]; used to cleanse the whole body, ease urine retention, and reduce wound infections
sbrul sha	Delivers baby, expels placenta [33]; and dissolves clots [37]
sdig srin	Downward circulation, prevents cramping, promotes uterine contractions, dilates cervix [33]; eases urine retention and is useful for various kidney disorder [37]
sga skya	Improves blood circulation and congestion, prevents clots [33]
star bu	Downward expelling of blood, prevents blood clots [34]; cools down fevers and provides pain relief, prevents blood clots (mostly used for women's disorders or lung problems) [33]

**Table 5:** Relevant therapeutic uses of Zhi Byed 11 ingredients in other traditional systems.

Tibetan name* (common name)	Tradition/region of use	Relevant therapeutic use within system/region
a ru Chebulic Myrobalan	Ayurvedic/Indian	Improving gastrointestinal motility, laxative, purgative, and cure for bleeding [38, 39]
bul tog Sodium, bicarbonate	Africa Herbal	Laxative [40]
lcum rtsa rhubarb	Traditional Chinese Medicine	Purgative, laxative [41]; to treat hemorrhage [30, 32]
	Ayurvedic	To treat hemorrhage [19, 21]
ol mo'se Himalayan mayapple	Traditional Chinese Medicine	Emmenagogue, uterine stimulant, and abortifacient [42]
sga skya ginger	Traditional Chinese Medicine	Increases gastric secretions, increases intestinal peristalsis [33]
	Ayurvedic	Treating constipation, nonspecific antispasmodic effect on smooth muscles [35, 43]
	Unani (Indian Traditional Greco-Arabic medicine)	Laxative [35]
	East African Herbal (Uganda)	Induction of uterine contractions [19]
star bu sea buckthorn	Traditional Asian Medicine (Mongolia and China)	Constipation gynecological diseases [44]

*Information not found for cong zhi, ma nu, rgya tsha, sbrul sha or sdig srin.
